# Recombinant amelogenin peptide TRAP promoting remineralization of early enamel caries: An *in vitro* study

**DOI:** 10.3389/fphys.2023.1076265

**Published:** 2023-01-23

**Authors:** Yaru Li, Yiwei Li, Qinghua Bai, Mingzhu Wen, Dandan Ma, Yisha Lin, Jinpu Chu

**Affiliations:** ^1^ The First Affiliated Hospital of Zhengzhou University, Zhengzhou, China; ^2^ College of Stomatology, Zhengzhou University, Zhengzhou, China

**Keywords:** peptide TRAP, enamel caries, remineralization, amelogenin, micro-CT

## Abstract

**Objective:** To explore the regulatory effect of recombinant amelogenin peptide TRAP on the remineralization of early enamel carious lesions.

**Methods:** Forty-eight bovine enamel blocks that prepared initial lesions *in vitro* were split at random into four groups for immersion treatment for 12 days: 1) remineralizing medium; 2) studied peptide 1 (consisting of the N- and C-termini of porcine amelogenin) + remineralizing medium; 3) studied peptide 2 (TRAP) + remineralizing medium; 4) fluoride + remineralizing medium. After demineralization and remineralization immersion, each specimen’s mean mineral loss and lesion depth were measured using micro-computed tomography (micro-CT). The changes in lesion depth (∆LD) and mineral gain (∆Z) were computed following remineralization. The enamel samples were then cut into sections and examined with polarized light microscopy (PLM). The cross-section morphology was observed by scanning electron microscopy (SEM). The crystal phase was analyzed by an X-ray micro-diffractometer (XRD). The calcium-binding properties were determined using isothermal titration calorimetry (ITC).

**Results:** Micro-CT analysis revealed a significant reduction in mineral loss in the four groups following the remineralization treatment (*p* < 0.05). The treatment with fluoride resulted in the greatest ∆Z and ∆LD, whereas the treatment with a remineralizing medium showed the least ∆Z and ∆LD among all groups. The ∆Z and ∆LD of the studied peptide 1 and studied peptide 2 groups were greater than those of the remineralizing medium group. However, there was no significant difference between the studied peptide 1 and studied peptide 2 groups (*p* > 0.05). All of the samples that the PLM analyzed had a thickening of the surface layer. A negative birefringent band changed in the lesion’s body. The SEM displayed that minerals were formed in all four groups of samples. The XRD results indicated that the products of remineralization of the studied peptide were hydroxyapatite crystals (HA). ITC showed that there were two binding modes between the calcium and peptide TRAP.

**Conclusion:** This study confirmed the potential of the recombinant amelogenin peptide TRAP as a key functional motif of amelogenin protein for enamel remineralization and provided a promising biomaterial for remineralization in initial enamel carious lesion treatment.

## Introduction

Dental caries in enamel is a unique disease of tooth hard tissue demineralized and dissolving by bacteria or other non-bacterial acidic attacks, and it is a major public health problem and a highly prevalent disease among the global population (Selwitz et al., 2007). After the enamel maturation, there is no longer a source for producing enamel structural units, and it is no longer available for natural enamel regeneration of damaged or destroyed tissue. Because of the lack of healing by cellular repair mechanisms, enamel caries tissues depend on the physicochemical process of repair. Strategies involving bioglass (Bakry et al., 2014), calcium phosphates (Cochrane et al., 2010), and fluoride (Majithia et al., 2016) have been applied for enamel regeneration. Fluoride, in particular, is widely used to treat dental caries. However, the mineral crystals in carious lesions that regenerate from fluoride are disordered. Furthermore, fluoride can potentially be harmful through overexposure (Li, 2003). The development of novel biomaterials that can safely promote carious lesion remineralization is an emerging approach. The enamel biomimetic remineralization strategy, in which the matrix material mediates the synthesis of HA through interactions with proteins or inorganic materials, is being extensively studied (Wang et al., 2017).

During the stage of enamel formation, ameloblast cells express and secrete enamel extracellular matrix proteins consisting principally of amelogenin and other proteins, including enamelin, ameloblastin, and proteinase (Bartlett et al., 2006). Amelogenin, which constitutes more than 90% of extracellular matrix proteins (Snead, 2003), mainly consists of three functional domains: a tyrosine-rich amelogenin peptide (TRAP), which is hydrophobic; a central domain (including proline and glutamine); and a hydrophilic C-terminus (Connelly et al., 2016). It has been demonstrated that the full-length amelogenin’s N- and C-terminal domains are crucial for the formation of the enamel mineral. The self-assembly into “nanospheres” or chain-like structures is mostly caused by the N-terminal domain. Apatite crystals must be directed toward parallel alignment *in vitro* by the C-terminal domain (Wang et al., 2020).

Because of its biomimetic mineralization capability, the enamel biomimetic remineralization strategy using amelogenin is being extensively researched. Diez-García et al. (2022) made amelogenin, ion exchange resin (containing calcium, phosphorus, fluorine, and zinc plasma), and artificial saliva into new products for bovine teeth, and found that it induced tooth remineralization to form fluorapatite, which is more acid-resistant and has the potential to promote long-term remineralization. Our previous study found that an amelogenin synthetic peptide (consisting of the N- and C-termini of porcine amelogenin) induces the remineralization of incipient enamel caries. As a mechanism for this action, the peptide may act as a regulatory factor and calcium ion carrier to direct ordered arrays of crystal formation (Chu et al., 2018).

In addition to full-length amelogenin, dental enamel contains a large number of peptide segments during development. TRAP is an important segment generated from full-length amelogenin through proteolytic clipping. A lectin-binding motif defined as “PYTSYGYEPMGGW” is present in TRAP (Paine and Snead, 2005). This lectin-binding property allows assembly into nanospheres according to the formation direction of ameloblasts, indicating that TRAP is responsible for amelogenin’s ability to self-assemble into “nanospheres” (Ravindranath et al., 2000). Domain “A” located on the TRAP segment, has full binding ability in isolation (Paine et al., 2001). The amelogenin-to-amelogenin interactions that cause nanosphere self-assembly require the A-domain, whereas the absence of the A-domain inhibits the self-assembly process (Dissanayake et al., 2020). The ability of TRAP to promote HA suggests that it may be able to self-assemble into a polyfunctional structure (Moradian-Oldak et al., 2002). Within the amelogenin peptide, the TRAP region offers the lone phosphorylation site (Se-16), which can interact with minerals such as calcium and phosphorus in the enamel formation stage (Le Norcy et al., 2011). Controlling the structural, compositional, mechanical, and crystallographic properties of enamel depends heavily on the phosphorylation of amelogenin. The biological regulation of the enamel mineralization process is reduced in the absence of amelogenin phosphorylation (Stifler et al., 2022).

In this study, the amelogenin peptide TRAP synthesized *in vitro* was used to explore its regulatory effect on the remineralization of incipient enamel caries, in order to clarify its function in amelogenin promoting remineralization of enamel caries. We hypothesized that TRAP is the pivotal functional motif of the natural amelogenin protein for the remineralization of dental enamel.

## Materials and methods

### Preparation of biomimetic peptides

**FIGURE 1:**
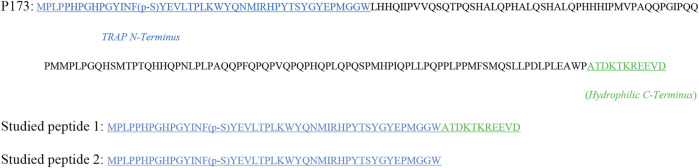
Full-length amelogenin P173 and synthesized peptide’s amino acid composition.

The peptides used in this study were produced by Synpeptide Co., Ltd. (Nanjing, China) using conventional solid-phase peptide synthesis, then purified and identified using HPLC and ESI-MS. They correspond to the amino acid sequences of porcine amelogenin P173. In Figure 1, studied peptide 1 was synthesized from the N- and C-termini of porcine amelogenin; studied peptide 2 is the peptide TRAP synthesized. Protein was first dissolved in deionized water to prepare a peptide stock solution (Chu et al., 2018). The peptide stock solution was stirred for 24 h on a stirrer at 4°C and then was placed in a refrigerator for at least 24 h.

### Sample preparation

**FIGURE 2:**
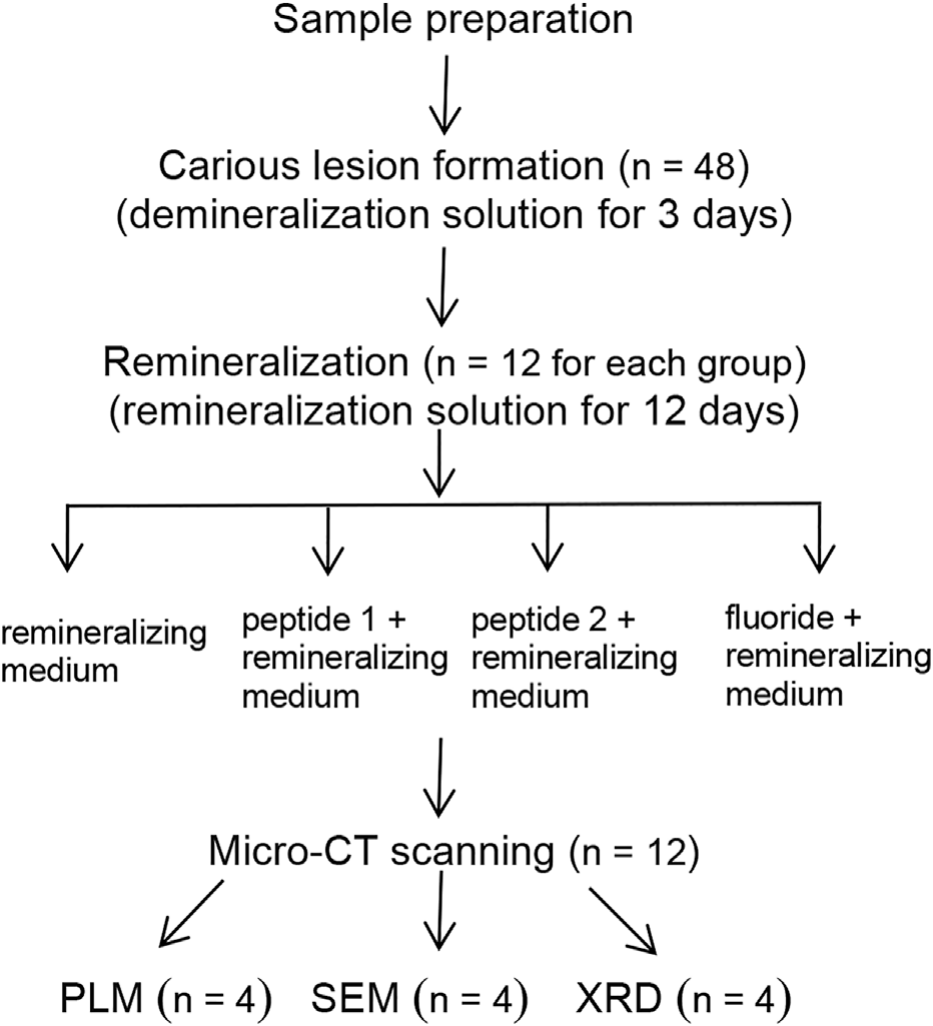
Flowchart of the study.

**FIGURE 3:**
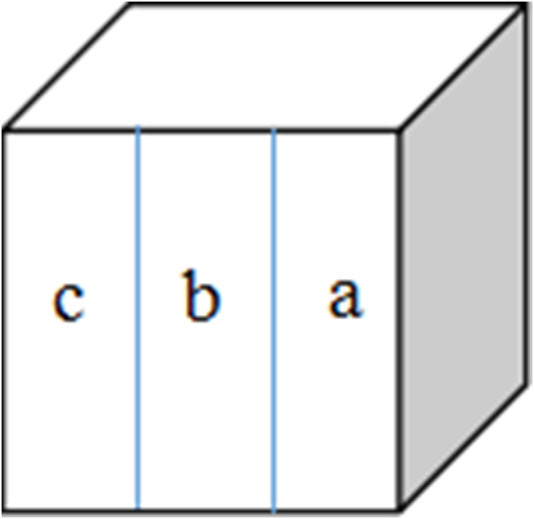
Specimen from the labial surface. Window **(a)** intact enamel reference, window **(b)** baseline caries, and window **(c)** test window.

Fresh bovine incisor teeth after extraction (approved by the First Affiliated Hospital of Zhengzhou University’s Ethics Committee: 2021-KY-1050-002) were employed. The crowns were separated from the roots and prepared into enamel blocks (3 mm × 3 mm × 2 mm) using a diamond-coated band saw under continuous water cooling (Struers Minitom; Struers, Copenhagen, Denmark). The study design is illustrated in Figure 2. Forty-eight enamel samples, free of cracks, cavities, chalky lesions, and enamel dysplasia, were selected under a stereological microscope. The enamel surfaces were then ground flat, polished with silicon carbide sandpaper of various grits (320, 400, 600, 800, 1200, 1500, 2000, and 2500 grades; Buehler Ltd.), and water-cooled to remove the enamel layer with a thickness of about 150 μm. Subsequently, the labial side of the enamel sample was divided into three regions, each with a size of 1 mm × 3 mm (a, b, and c; Figure 3). The reference window with intact enamel was window a, the baseline for caries was window b, and the test window was window c.

### Carious lesion formation

The surfaces of the samples were all covered with acid-resistant nail varnish, except for windows b and c. To create artificial carious lesions, forty-eight blocks were immersed in 192 ml of demineralization solution (5.0 mM NaN_3_, 50 mM acetic acid, 2.2 mM Ca (NO_3_)_2_, 2.2 mM KH_2_PO_4_, and 0.2 ppm NaF; pH was adjusted to 4.5 using 5 mM KOH), at 37°C for 3 days with 100 RPM continuous magnetic stirring (Ten and Duijsters, 1983). The demineralization solution was replenished every 24 h. After demineralization, all samples were rinsed with deionized water and dried naturally.

### Remineralization treatment

All enamel specimens were divided at random into four different remineralizing treatments (12 specimens per group): group A, remineralizing medium; group B, 100 μg/ml studied peptide 1 + remineralizing medium; group C, 100 μg/ml studied peptide 2 + remineralizing medium; group D, 2 ppm fluoride + remineralizing medium. Specimens from each group were immersed in 18 ml of the corresponding remineralizing medium (0.9 mM KH_2_PO_4_, 1.5 mM CaCl_2_, 20 mM HEPES, 130 mM KCl, and 1 mM NaN_3_; the pH was adjusted to 7.0 with 5 mM KOH), at 37 °C for 12 days with 100 RPM continuous magnetic stirring (Almqvist and Lagerlof, 1993). The remineralizing medium was replenished once per day. The enamel samples were washed with deionized water and air-dried after remineralization treatment.

### Micro-CT scanning

Each specimen’s mineral density (MD) and lesion depth (LD) were assessed using a micro-CT system (Skyscan 1272, Bruker, Germany) after remineralization. The parameters were set to a voltage of 60 kV, a current of 160 μA, and a resolution of 8.5 μm. After scanning, the profiles of all samples were reconstructed using the NRecon software in Skyscan 1272. For MD calibration, a collection of mineral reference phantoms, including three HA disks (50, 250, and 750 mg/cm^3^), was scanned.

Each specimen’s sound, baseline, and test windows were all examined at their respective centers in a volume of interest (VOI) of 300 μm × 300 µm. By assuming a sound enamel with a maximum MD of 100 vol%, the MD profile was transformed into a relative MD. The data for the reconstruction of the three-dimensional image of the enamel blocks were collected with a resolution of 1024 × 1024 and voxel sizes of 8.5 μm isotropic. The average MD value was calculated from the MD of the sample. In addition, the three-dimensional VOI of 100 μm × 100 μm × 100 μm was randomly selected at the center of the three regions. The average densities were expressed as MDa, MDb, and MDc, respectively. The remineralization rate (%R) was calculated using the following formula:

$$\%R=\frac{∆Z}{∆Zde}\times 100$$

ΔZde (MDa-MDb) is mineral loss after demineralization; ΔZre (MDa-MDc) is mineral loss after remineralization; and ∆Z (∆Zde-ΔZre) is mineral gain after remineralization.

A coronal image of each sample was obtained. To obtain the lesion depths of the baseline and test windows, the assessments were randomly selected at the center of each coronal image of the sample. The average values were LDb and LDc, and the changes in lesion depth (ΔLD) before and after remineralization can be obtained, ΔLD = LDb-LDc.

### X-ray diffractometer testing

Enamel samples were first embedded in acrylic resin and then longitudinally cut in half and polished to achieve a flat surface and a sample thickness of 100 µm. These sections were analyzed using an X-ray micro-diffractometer (D8 Discover; Bruker, Germany). The data were collected in the scanning range of 20°–65° under the conditions of Cu target Kα radiation, tube voltage of 40 Kv, and tube current of 40 mA. The data were analyzed using Jade 6.0 software. Then the obtained XRD pattern was compared with a standard diffraction card (JCPDS 09-0432). (n = 4 for each group).

### SEM morphology

Enamel samples were first embedded in acrylic resin and then transversely cut in half. Then the samples were dried and sputter-coated with gold. The cross-section morphology was observed by SEM (Sigama, 500; Zeiss, Germany). (n = 4 for each group).

### Polarized light microscopy examination

The enamel specimens were embedded in acrylic resin. From the center of each specimen, thin, plane, parallel pieces that were about 100 μm thick were cut and polished. Glass microscope slides were used to mount the slices, which were assessed using a polarized light microscope (Axio Scope Al, Zeiss, Germany). (n = 4 for each group).

### Calcium-binding test

The thermodynamics of Ca^2+^ binding to peptide 2 were studied using isothermal titration calorimetry (TA Instruments, USA). At 37°C and 350 rpm, ITC measurements were carried out in a standard volume of nanoITC. Water was put into the reference cell. A 10 mM HEPES solution containing peptide 2 was created (pH = 7.0). A solution of peptide 2 in HEPES at 0.42 mM received an injection of 12.5 mM CaCl_2_ (10 mM). Twenty-five separate injections of 2 µL each were used to titrate CaCl_2_ into the peptide solution at intervals of 180 s. The heat generated by calcium ions binding to TRAP per injection was measured and was displayed as differential power (µcal s^−1^) vs. time (min). The area under each injection peak was integrated and presented as kcal mol^−1^ of the injectant vs the molar ratio of [Ca^2+^] ÷ [TRAP]. In a control experiment, the same amount of CaCl_2_ was titrated into HEPES (10 mM), and the results were then deducted from the raw heat of the reaction to get the effective heat of binding. ITCRun data collection software used the ITC one-set of sites model to calculate the thermodynamic values of calcium binding to peptide 2. Calcium binding properties are expressed as affinity constant, dissociation constant, enthalpy, and entropy.

### Data analysis

Statistical analysis of the experimental data was performed using IBM SPSS 21.0 (IBM, Chicago, IL, United States). All data are presented as the mean and standard deviation. The ∆Z, ΔLD, and %R were determined by one-way ANOVA followed by post hoc LSD. Multiple comparisons were used to analyze the micro-CT data, and the test level was *α* = 0.05.

## Results

### Micro-CT data

**FIGURE 4:**
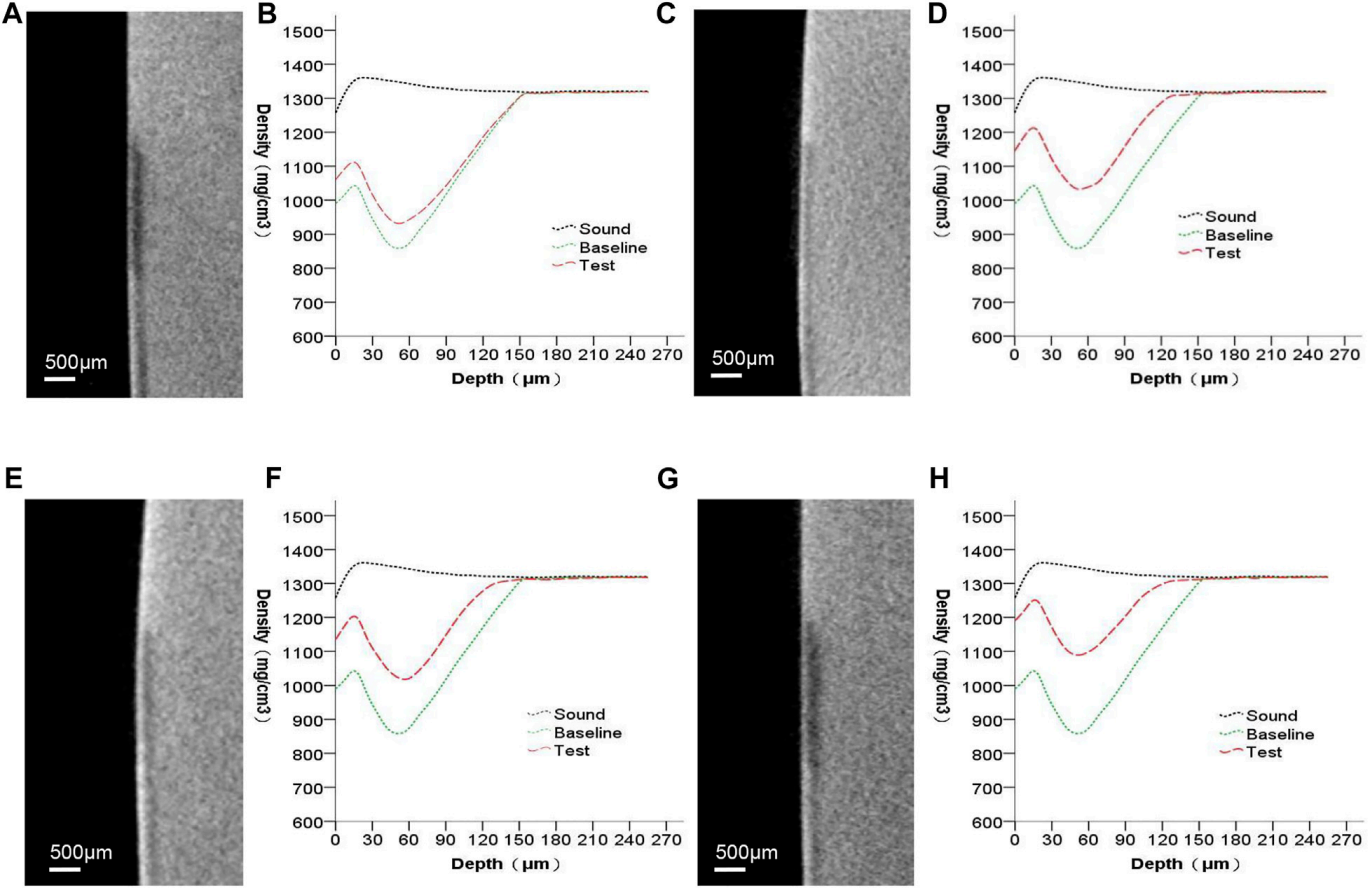
Micro-CT images of all groups. **(A,B)** remineralizing medium group; **(C,D)** studied peptide 1 group; **(E,F)** studied peptide 2 group; **(G,H)** fluoridate group.

Figure 4 presents the micro-CT 2D image of the enamel sample (a,c,e,g) and the profiles of mineral density against depth from the objects’ surfaces (b,d,f,h). Images (a,c,e,g) include the sound, baseline, and test windows (from top to bottom). Subsurface lesions were confirmed using baseline profiles. The mineral density increased throughout the test profiles. The only medium (group A) that remineralized minerals showed mostly enhanced mineral density at 0–60 µm. At a depth greater than 60 µm for the baseline and test windows in the remineralizing media, the mineral density gradually converged. Except for the group that received only the remineralizing media, the other groups affected both the surface layer and the deeper layer of the lesion.

**FIGURE 5:**
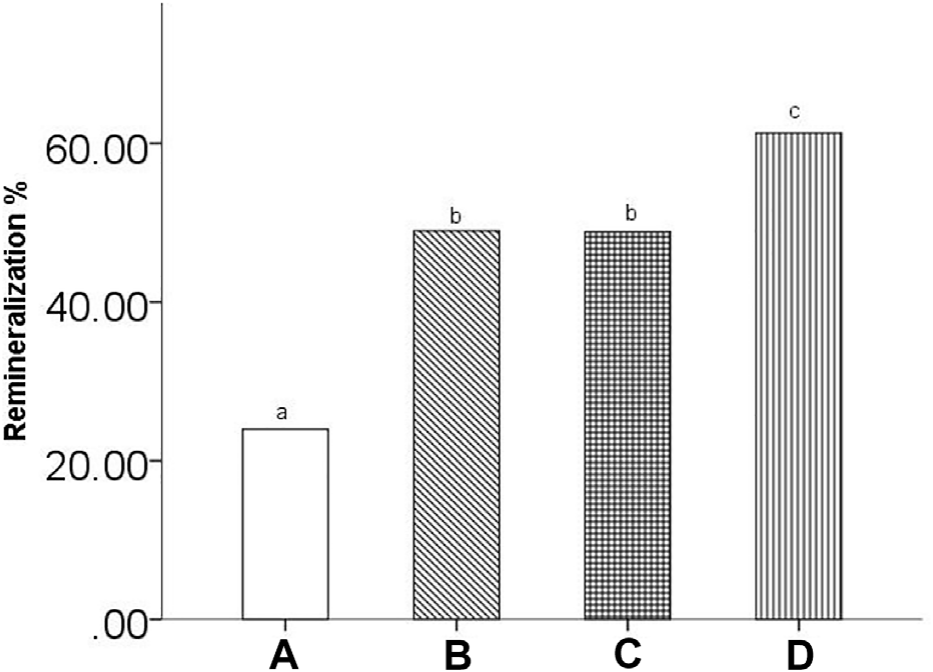
The remineralization rate of all groups. **(A)** remineralizing medium group; **(B)** studied peptide 1 group; **(C)** studied peptide 2 group; **(D)** fluoridate group. The same letter above the bar chart shows that no difference is statistically significant (*p* > 0.05).

Figure 5 shows the %R of all groups. The mean %R ± SD values were 23.96% ± 6.16, 48.96% ± 6.06, 48.80% ± 8.95, and 61.29% ± 7.40 for the remineralizing medium, peptide 1, peptide 2 and fluoridate remineralizing medium groups, respectively. One-way ANOVA statistical analysis showed significant differences between the treatment groups (*p* < 0.05). Additionally, the LSD post hoc test for multiple comparisons indicated significant differences in %R within the treatment groups. Except for the peptide 1 and peptide 2 groups (*p* > 0.05), all groups had significant differences (*p* < 0.05).

Table 1 displays the ∆Z from the MD profiles of all groups after remineralization. The data are described using the mean ± standard deviation. Results of the one-way ANOVA revealed that all differences in the medium treatments were statistically significant (*p* < 0.05). Additionally, the MD between the treatment groups varied significantly according to multiple comparisons using the LSD post hoc test. However, there was no significant difference between the studied peptide 1 and peptide 2 groups (*p* > 0.05).

**TABLE 1:** Results of mineral loss and gain of the enamel samples.

Group	∆Zde (mg/cm^3^)	∆Zre (mg/cm^3^)	∆Z (mg/cm^3^)
remineralizing medium	319.75 ± 36.76	243.42 ± 36.66	76.33 ± 20.02^a^
studied peptide 1	311.42 ± 25.92	159.92 ± 30.15	151.50 ± 14.05^b^
studied peptide 2	309.50 ± 40.61	160.91 ± 45.62	148.58 ± 19.80^b^
Fluoridate	313.33 ± 35.29	123.17 ± 35.64	190.17 ± 14.86^c^

**Note** the same letters (a though c) across groups indicate no difference in the same table (*p* > 0.05). ΔZde is mineral loss after demineralization; ΔZre is mineral loss after remineralization; ΔZ is mineral gain after remineralization.

Table 2 illustrates the ΔLD in the profiles of each group after demineralization and remineralization. The data are described using the mean ± standard deviation. The one-way ANOVA identified a statistically significant difference in ∆LD among the test groups (*p* < 0.05). The LSD post hoc multiple comparison analysis showed that the ∆LD were greatest for the fluoridate remineralizing medium group and the least for the mineralizing medium group. There was no significant difference in the ∆LD between the studied peptide 1 and peptide 2 groups (*p* > 0.05).

**TABLE 2:** Results of lesion depth.

Group	LDb (μm)	LDc (μm)	∆LD (μm)
remineralizing medium	87.12 ± 5.94	68.00 ± 4.70	19.13 ± 4.02^d^
studied peptide 1	75.30 ± 6.56	45.34 ± 4.00	29.98 ± 4.28^e^
studied peptide 2	82.88 ± 9.05	54.53 ± 7.56	28.33 ± 5.94^e^
Fluoridate	89.01 ± 7.98	47.22 ± 9.31	41.78 ± 6.30^f^

**Note** the same letters (d, e, f) across groups indicate no difference (*p* > 0.05). The lesion depth is calculated from the subsurface. LDb is the lesion depths after demineralization; LDc is the lesion depths after remineralization; ΔLD is the changes in lesion depth.

### Polarized light microscopy examination

**FIGURE 6:**
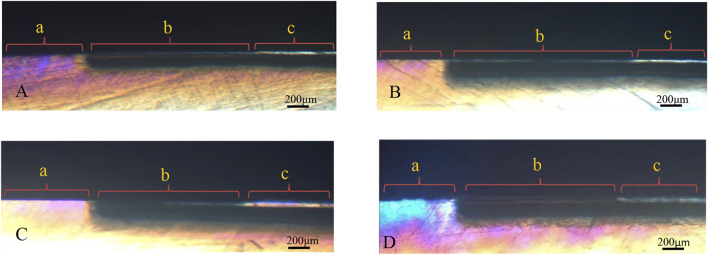
PLM pictures for the groups. **(A)** remineralizing medium group. **(B)** studied peptide 1 group. **(C)** studied peptide 2 group. **(D)** fluoridate remineralizing medium group.

Figure 6 shows the polarized light profiles of all groups. All demineralized regions with increased tissue porosity appeared to be positively birefringent when the slices were analyzed by PLM after imbibition in water (RI = 1.33). The enamel lesions had an intact surface layer (the area of negative birefringence) above positively birefringent lesion bodies. In all groups, except for the remineralizing medium group, the thickness of the surface layer and density clearly increased after remineralization. For the remineralizing medium group, a small increase in the density of the surface layer was observed.

### SEM morphology

**FIGURE 7:**
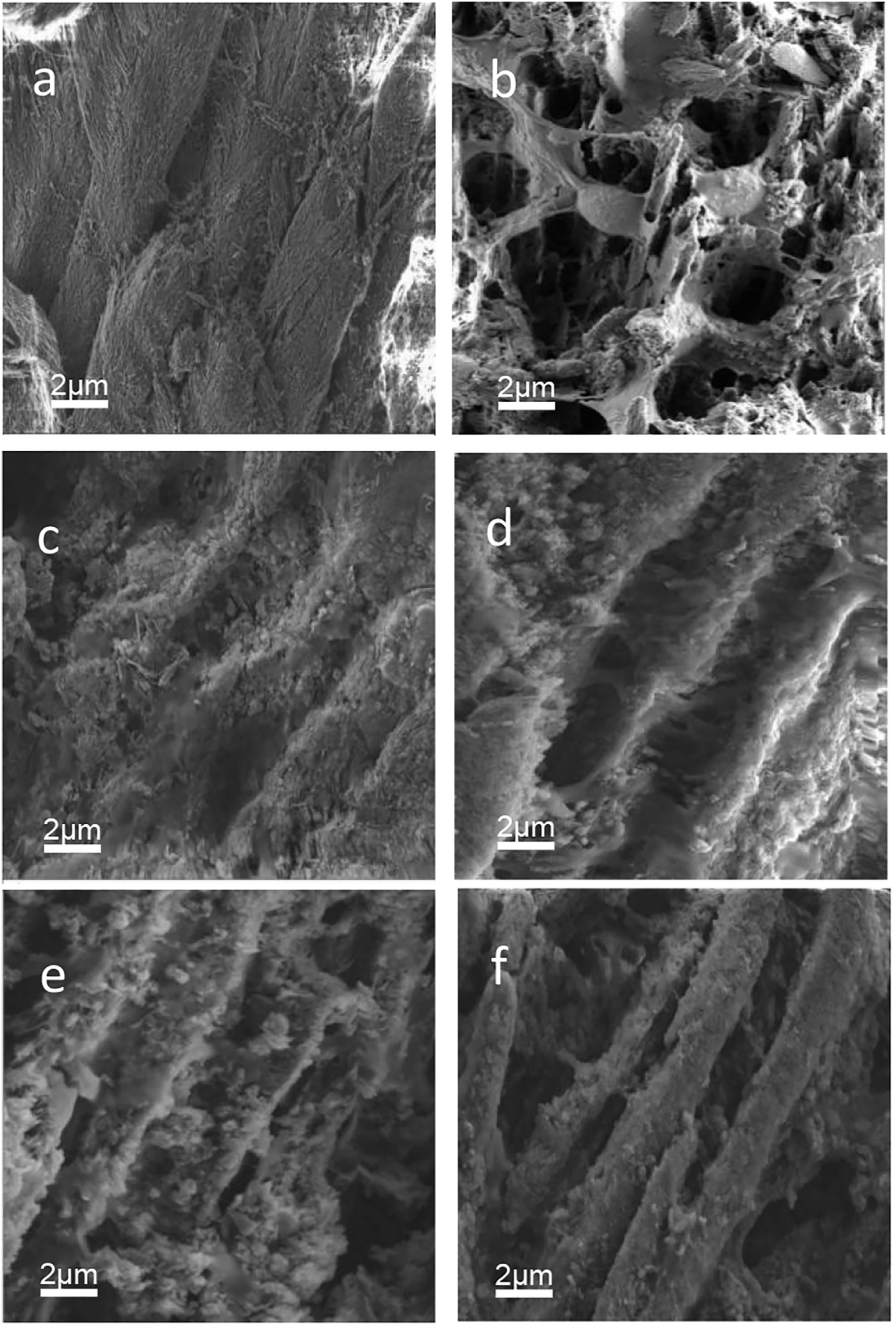
SEM pictures. **(a)** sound enamel. **(b)** demineralized enamel. **(c)** remineralizing medium group. **(d)** peptide 1 group. **(e)** peptide 2 group. **(f)** fluoridate remineralizing medium group.

Figure 7 presents the cross-section SEM images. Well-aligned enamel rods can be obversed in sound enamel 1). After 3 days of demineralization, the enamel rods of sound enamel were significantly destroyed 2). The remineralizing medium group formed mineral layers on its cross-section 3). The imprints of enamel rods are visible as shallow depressions in the cross-section image of peptide 1 group 4). The cross-section image of the peptide 2 group also shows similar inter-rod gaps. In addition, some minerals adsorb on the surface 5). The fluoridate remineralizing medium group obtained the most obvious enamel rods 6).

### X-ray diffractometer spectra

**FIGURE 8:**
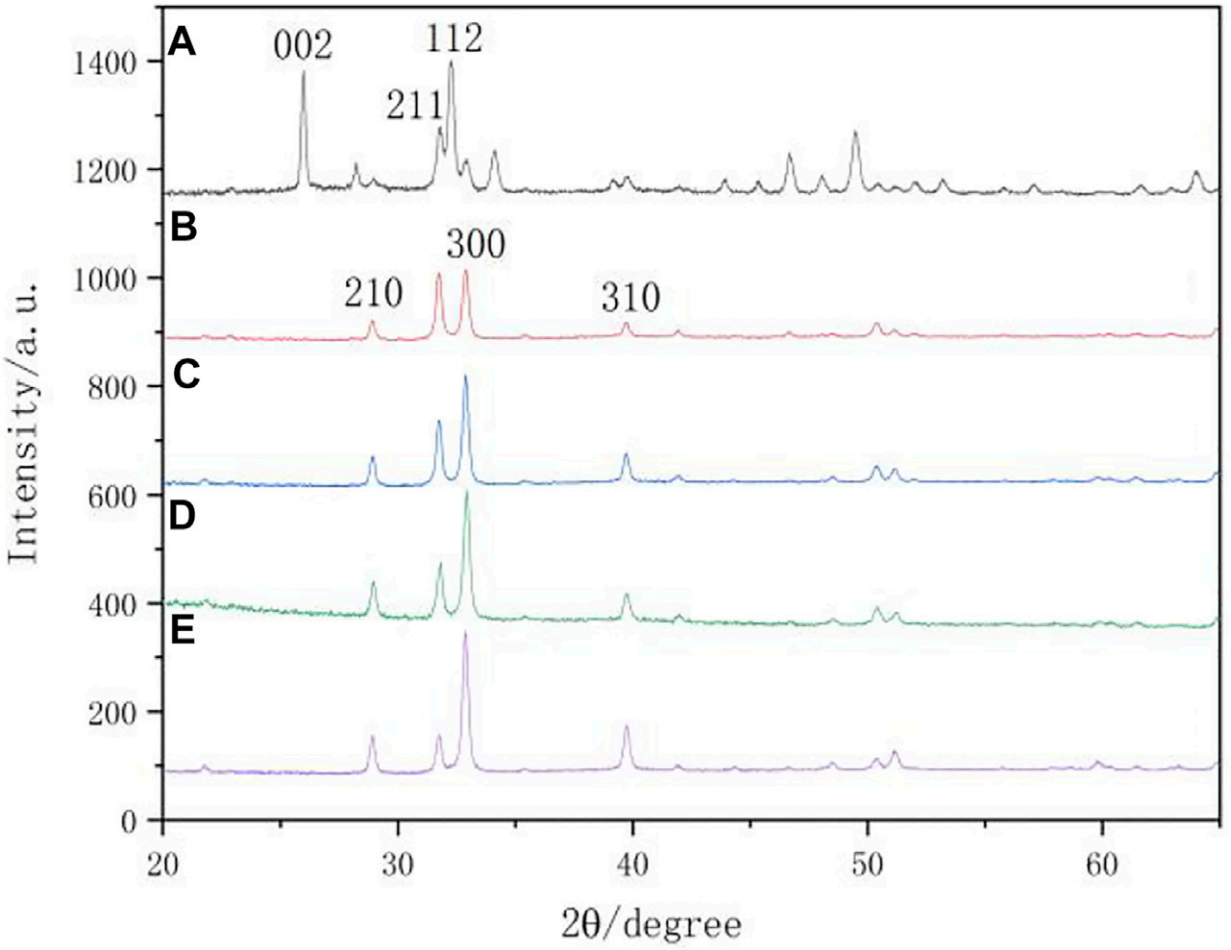
XRD spectra. **(A)** native enamel; **(B)** remineralizing medium; **(C)** studied peptide 2; **(D)** studied peptide 1; **(E)** fluoridate.

Figure 8 shows the XRD spectra of the surfaces of the natural enamel and enamel disks after remineralization. The characteristic peaks displayed on the remineralization surface of each group were compared with those of the standard card (JCPDS 09-0432). The diffraction peaks at approximately 29.0° (210), 31.8° (211), 32.9° (300), and 39.8° (310) are consistent with the characteristic crystalline peaks of HA, indicating that the substance generated after remineralization is HA. The (210), (211), and (300) peaks of XRD that were found on the remineralizing media indicate that crystalline HA started to form, but only in a small amount. In comparison with the remineralizing medium group, the peaks of (210) and (300) were enhanced, indicating that the remineralization of other groups was suppressed.

### Calcium-binding effects

**FIGURE 9:**
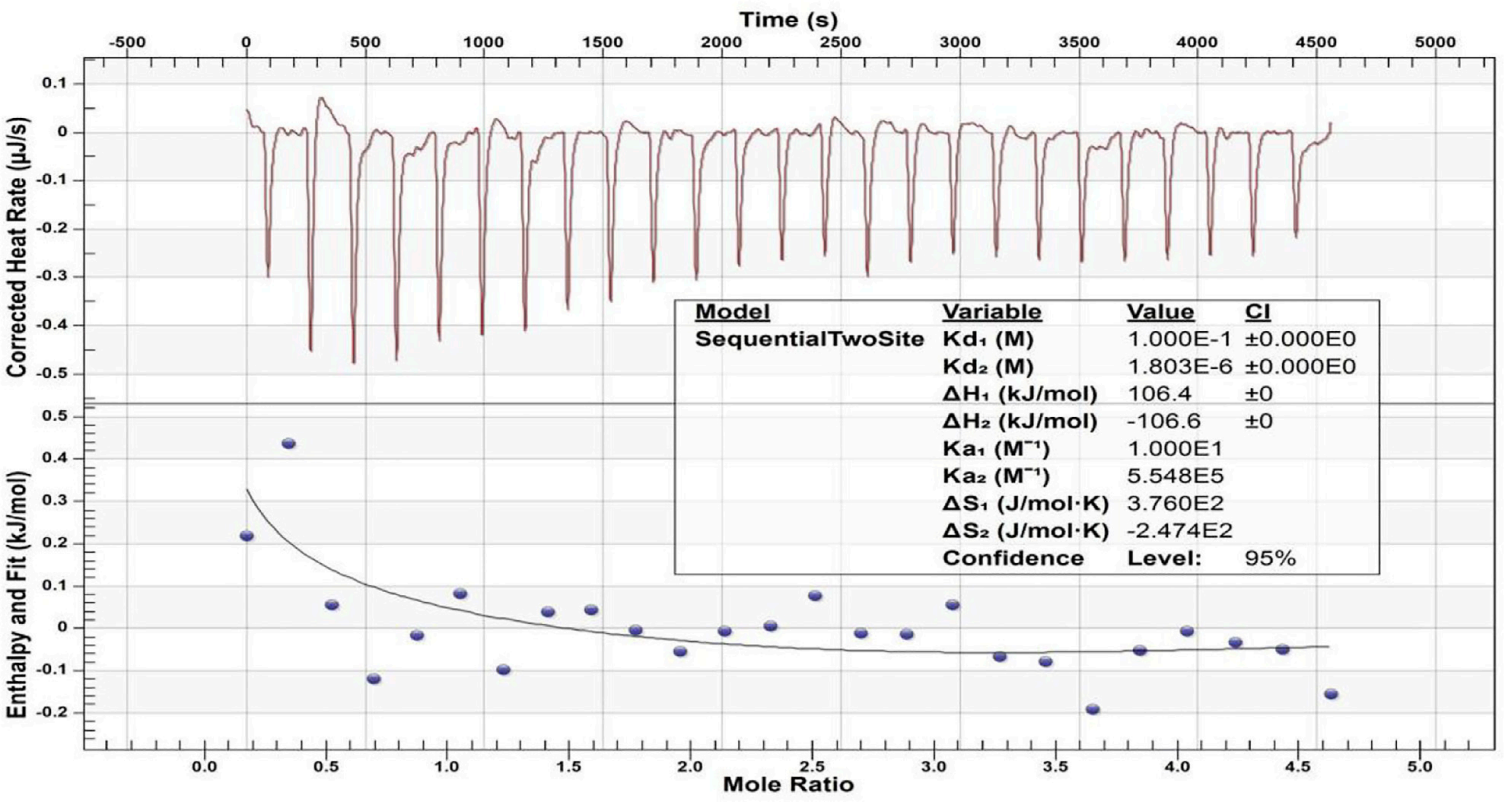
ITC-based Ca^2+^ binding study of peptide 2. The top panel displays the raw titration data measured, and the bottom graph provides integrated heat data following corrections of dilution heat against molar ratio of Ca^2+^/peptide 2.

Figure 9 displays the heat generation spectrogram and effective binding isotherms between the calcium ions and peptide 2. The downward curve indicated an endothermic reaction. The change in the heat value of the binding isotherm was stable and abnormal in the heat generation spectrogram from the fifth drop in the binding isotherm graph, indicating binding saturation. The different thermodynamic values of the affinity constant indicated two distinct types of binding events between Ca^2+^ and peptide 2. A thermodynamic value analysis revealed that the second affinity constant (Ka_2_ = 5.548 × 10^5^ M^−1^) of the Ca^2+^ and peptide was higher than the first affinity constant (Ka_1_ = 10 M^−1^), and the first dissociation constant (Kd_1_ = 1.000 × 10^–1^ M) was lower than the second dissociation constant (Kd_2_ = 1.803 × 10^–6^ M), suggesting that Ca^2+^ binding to peptide 2 may be easier for the second type of binding event. The values of enthalpy (ΔH_1_ and ΔH_2_) were 106.4 kcal mol and -106.6 kcal mol, respectively, and entropy (ΔS_1_ and ΔS_2_) were 3.760 × 10^2^ cal mol^−1^ °C^−1^ and -2.474 × 10^2^ cal mol^−1^ °C^−1^, respectively, for Ca^2+^ and TRAP.

## Discussion

The results of this study indicated the potential of the synthetic peptide TRAP to promote the remineralization of incipient enamel caries. Furthermore, the trend of remineralization of TRAP was the same as that of the peptide consisting of the C- and N-termini of amelogenin. Therefore, our results provide support for the hypothesis that the synthesized peptide TRAP should be the key functional motif of native amelogenin for remineralization.

A miniature type of medical CT scanning called micro-CT has a spatial resolution of a few micrometers (Besinis et al., 2014). Micro-CT is an advanced technique that can quantify the mineral loss and lesion depth of enamel with two- and three-dimensional information (Zhang et al., 2021). The pixel resolution is accurate to within 1 μm. The technology of micro-CT has been considered a substitute for non-destructively quantitatively characterizing surface topography.

The mineral loss was significant (Figure 4; a,c,e,g) and comparatively stable (Table 1; ∆Zde) among the four experimental groups after demineralization, according to the micro-CT results. Subsurface lesions with intact surface layers were observed in all groups of samples after demineralization in the PLM micrographs (Figure 6). These results suggest that the artificial carious lesions in the samples were successfully prepared and were similar to natural carious lesions. This finding was also reported by Huang et al. (2011). Protein molecules have been shown to promote remineralization in enamel lesions. Leucine-rich amelogenin peptide (LRAP), which Zhong et al*.* (2021) self-assembled with TRAP, was found to effectively stabilize calcium and phosphorus ions in amorphous calcium phosphate (ACP) and direct the growth of ACP along its C-axis into bundles of HA crystals. In this study, the remineralization effect of the peptide 2 group was higher than that of the remineralizing medium group (Figure 5; Table 1; Table 2), indicating that peptide 2 promotes the remineralization of enamel caries. Amelogenin is well known for regulating HA growth and enamel mineralization. The N-terminus is an important domain of amelogenin for assisting mineralization (Dissanayake et al., 2020). Through its tyrosine enrichment segment (TRAP) on the N-terminus, amelogenin interacts with calcium and phosphorus ions, stabilizing them into an amorphous form (Yan et al., 2022). There was no significant difference in remineralization between the peptide 1 and peptide 2 medium treatment groups. This suggests that the peptide TRAP can be used as a functional fragment of amelogenin to promote the remineralization of incipient enamel caries.

The cross-section characteristics of six groups of SEM images (Figure 7) showed significant differences. The remineralization medium at pH 7.0 in this study was equivalent to artificial saliva. When remineralizing medium is used alone, the treatment results in mineralized layers due to the repair ability of the demineralized enamel in artificial saliva. However, the limited Ca^2+^ ions in the remineralization medium restrained the remineralization. As a result, the enamel rods are not obvious (Zhang et al., 2014). The peptide 1 and peptide 2 groups showed a more ordered structure of remineralization. Matsumoto et al. (2006) demonstrated that the peptide could attract Ca^2+^ and PO_4_^3-^ ions, thereby maintaining topical supersaturation and eventually catalyzing newly formed mineralization. This supports the results of this study. The morphology of the mineral formations in the peptide group and fluoridate remineralizing medium group is different. In the fluoridate remineralizing medium group, the new enamel rod structures are closest to those of the sound enamel. The mechanism of fluoride action is through decreasing the dissolution of the newly formed apatite.

X-ray micro-diffraction can be used to get more specific information (Xue et al., 2008). So it was used to detect the surface of the caries lesions and remineralization areas of all specimens and to further explore the crystal phase structure of the peptide TRAP synthesized for regulating the remineralization of enamel caries lesions in this study. Previous studies have demonstrated that the product of an amelogenin-derived peptide applied for remineralization was HA (Ruan et al., 2014; Peng et al., 2021). The N-terminal tyrosine segment of amelogenin was extracted from LRAP and synthesized into a biomimetic enamel matrix protein using non-amelogenin analogs. HA is formed on the surface of enamel caries and has mechanical properties similar to those of natural enamel (Fang et al., 2021). As shown in the XRD pattern (Figure 8), the crystals of the natural enamel were HA. Characteristic diffraction peaks of HA appeared on the surface of the samples after remineralization. From the intensity of the HA peak in this study, it was confirmed that the crystallinity of the HA crystals formed for the peptide 2 closely resembled that of the peptide 1. HA crystals in natural enamel have the characteristic of preferential growth along the C axis (Figure 8A). However, the HA crystals formed by the enamel samples after remineralization in each group did not show an obvious peak (002) that preferentially grew along the C axis. This phenomenon may be due to the different mechanisms of enamel biomineralization and remineralization *in vitro*. Biomineralization is influenced by various factors, including biological agents. However, this study was conducted under *in vitro* conditions, which lacked biological gene regulation.

Our previous study confirmed that our synthetic peptide (consisting of the N- and C-termini of porcine amelogenin) can bind to Ca^2+^ with a high affinity. The negatively charged of N-terminus (phosphorylated serine residues) was primarily involved in the binding (Chu et al., 2018). In the present study, the values of the thermodynamic parameters changed (Figure 9), with ΔH_1_ > 0, ΔS_1_ > 0, and TΔS_1_ > ΔH_1_, indicating that the binding was mostly driven by entropy and that hydrophobic interaction was the primary force (Ross and Subramanian, 1981). Therefore, the first type of binding event was mediated by the hydrophobic interaction of peptide 2. The second values of the parameter changes with ΔH_2_ < 0 and ΔS_2_ < 0 indicate that the binding is mediated by the hydrogen bond and van der Waals force between the calcium ions and peptide 2. The dissociation constant (Kd_1_ > Kd_2_) and affinity constant (Ka_1_ < Ka_2_) indicate the interaction of Ca^2+^ and TRAP mainly by hydrogen bonding and van der Waals forces. This study supports evidence from previous observation (Wang et al., 2012). The studied peptide 2 can bind to calcium ions, which should be related to the negatively charged phosphate group in the amino acid sequence of TRAP. Thus, from the micro-CT and PLM results obtained in this study, we believe that peptide TRAP can operate as a Ca^2+^ ion carrier, delivering Ca^2+^ ions from the remineralizing solution to the deeper layers of caries lesions and promoting remineralization by giving the Ca^2+^ needed for crystal repair.

## Conclusion

The application of a surface layer of peptide TRAP solution to enamel lesions improved mineral gain, decreased lesion depth, and produced HA crystals. The mechanism of action as a calcium ion carrier supports the action of the recombinant amelogenin peptide TRAP in the regeneration of enamel tissue. This study provided encouraging findings for future conservative management of early enamel lesions. Further research should be undertaken to investigate the microscopic crystal structure of the products of enamel caries remineralization induced by the TRAP peptide. And *in situ* and animal experimental models are needed to further explore the remineralization of the peptide TRAP.

## Data Availability

The original contributions presented in the study are included in the article/supplementary materials, further inquiries can be directed to the corresponding author.
